# 1931. Real-world evaluation of adequacy of empiric and definitive therapy in carbapenem susceptible and non-susceptible Enterobacterales and *Pseudomonas aeruginosa* by time to culture result: A multicenter analysis

**DOI:** 10.1093/ofid/ofad500.091

**Published:** 2023-11-27

**Authors:** Todd Riccobene, ChinEn Ai, Kalvin Yu, Sara Gregory, John L Lock, Brooke Kim, Vikas Gupta

**Affiliations:** AbbVie Inc, Madison, New Jersey; BD - Becton, Dickinson and Company, Atlanta, Georgia; Becton, Dickinson and Company (BD), Franklin Lakes, New Jersey; Becton, Dickinson and Company, Franklin Lakes, New Jersey; AbbVie Inc, Madison, New Jersey; AbbVie, Madison, New Jersey; Becton, Dickinson and Company (BD), Franklin Lakes, New Jersey

## Abstract

**Background:**

Infections caused by multi-drug resistant (MDR) Gram-negative pathogens are increasingly problematic in healthcare settings and are associated with worse clinical outcomes in critically ill patients. We conducted a multicenter real-world evaluation of culture turn-around time (TAT), associated inadequate empiric (IET) and definitive therapy (IDT) in carbapenem susceptible (Carb-S) and non-susceptible (Carb-NS) Enterobacterales (ENT) and *P. aeruginosa* (PSA).

**Methods:**

Hospitalized adults (≥ 18 years old) with facility reported antibiotic susceptibility from 2018-2022 across 161 facilities (BD Insights Research Database, Franklin Lakes, NJ) were identified for non-contaminant Carb-S/Carb-NS ENT and PSA across respiratory, blood, urine, intra-abdominal, skin/wound, and other sources. We evaluated antibacterial therapy as IET (prior to first susceptibility result) and IDT (48hrs post first susceptibility result & not discharged) by culture TAT (date/time first susceptibility results – date/time culture collection).

**Results:**

We identified 345,245 ENT of which 1.8% (6,174) were Carb-NS and 60,143 PSA of which 17.2% (10,357) were Carb-NS. IDT was lower than IET in Carb-S ENT (12.9% vs. 5.1%), Carb-NS ENT (53.5% vs. 25.2%), Carb-S PSA (28.4% vs. 11.0%), and Carb-NS PSA (53.5% vs. 26.0%) pathogen results. IDT was also lower than IET at each 12-hour increment of availability of susceptibility results (**Figure 2**) and overall IDT was 25.2% and 26.0% in Carb-NS ENT and Carb-NS PSA, respectively. Median time to positive susceptibility result was longer in Carb-NS ENT vs. Carb-S ENT (67 vs. 53 hrs) and longer in Carb-NS PSA vs. Carb-S PSA (68 vs. 62 hrs) (**Table 1, Figure 1**).

**Figure:**
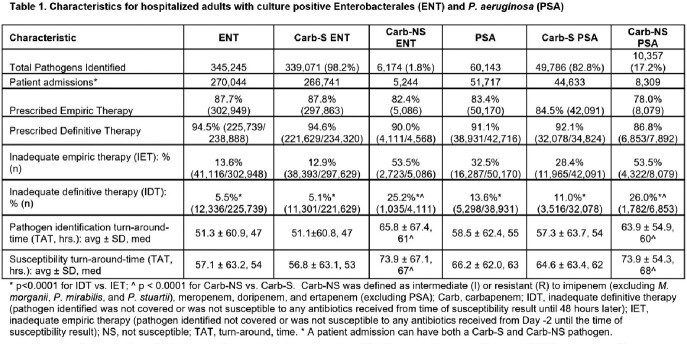

**Figure:**
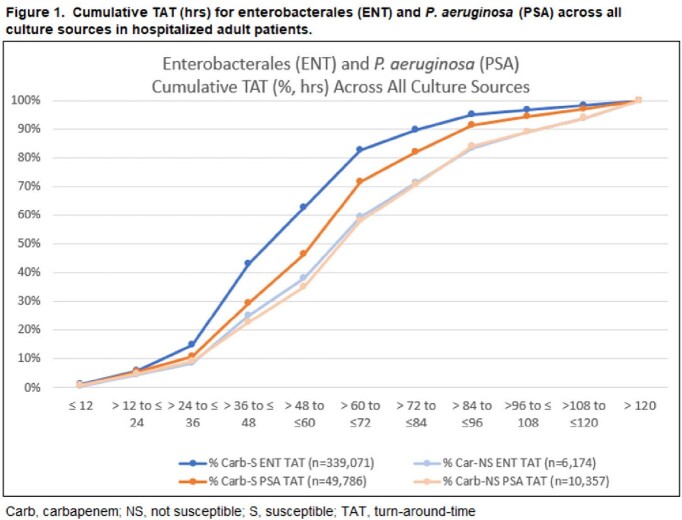

**Figure:**
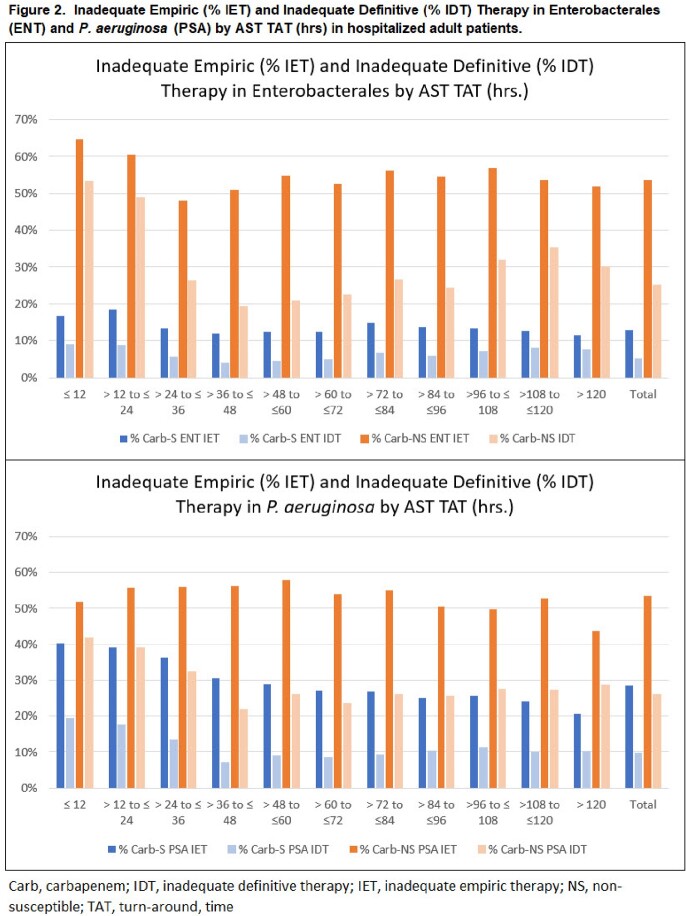

**Conclusion:**

There was a reduction in inadequate therapy prescribed once cultures were available (IET to IDT) overall and across each of the 12-hr TAT to susceptibility results periods evaluated in Carb-S and Carb-NS ENT and PSA positive cases. Both IET and IDT were higher in patients with either Carb-NS ENT or Carb-NS PSA than Carb-S patients. However, more than 25% of patients with Carb-NS ENT/PSA were prescribed IDT within 48 hours of a susceptibility result. These data support efforts to improve TAT to susceptibility results and can inform protocols and workflow that guide empiric and definitive therapy.

**Disclosures:**

**Todd Riccobene, PhD**, AbbVie: Employee salary|AbbVie: Stocks/Bonds **ChinEn Ai, MPH**, Becton, Dickinson and Company: Employee **Kalvin Yu, MD, FIDSA**, BD: Stocks/Bonds **Sara Gregory, PhD**, Becton, Dickinson and Company: Employee **John L. Lock, PharmD**, AbbVie: Employee|AbbVie: Stocks/Bonds **Brooke Kim, RN, BSN, MSM**, AbbVie: Employee|AbbVie: Stocks/Bonds **Vikas Gupta, PharmD**, Becton, Dickinson and Company (BD): Employee of BD|Becton, Dickinson and Company (BD): Stocks/Bonds

